# A Grape Juice Supplemented with Natural Grape Extracts Is Well Accepted by Consumers and Reduces Brain Oxidative Stress

**DOI:** 10.3390/antiox10050677

**Published:** 2021-04-26

**Authors:** Miriam Bobadilla, Carlos Hernández, María Ayala, Ixone Alonso, Ana Iglesias, Josune García-Sanmartín, Eduardo Mirpuri, José Ignacio Barriobero, Alfredo Martínez

**Affiliations:** 1 Oncology Area, Center for Biomedical Research of La Rioja (CIBIR), Piqueras 98, 26006 Logroño, Spain; mbobadilla@riojasalud.es (M.B.); jgarcias@riojasalud.es (J.G.-S.); emirpuri@riojasalud.es (E.M.); 2 Food Technology and Innovation Center of La Rioja (CTIC-CITA), 26315 Alesón, Spain; chernandez@cticcita.es (C.H.); mayala@cticcita.es (M.A.); ialonso@cticcita.es (I.A.); aiglesias@cticcita.es (A.I.); jibarriobero@cticcita.es (J.I.B.)

**Keywords:** acute oxidative stress, neurodegenerative diseases, red grape polyphenol extract, MecobalActive^®^, restraint stress

## Abstract

Neurodegenerative diseases pose a major health problem for developed countries. Stress, which induces oxidation in the brain, has been identified as the main risk factor for these disorders. We have developed an antioxidant-enriched drink and have examined its protective properties against acute oxidative stress. We found that addition of red grape polyphenols and MecobalActive^®^ to grape juice did not provoke changes in juice organoleptic characteristics, and that the pasteurization process did not greatly affect the levels of flavonoids and vitamin B12. Out of all combinations, grape juice with red grape polyphenols was selected by expert judges (28.6% selected it as their first choice). In vivo, oral administration of grape juice supplemented with red grape polyphenols exerted an antioxidant effect in the brain of stressed mice reducing two-fold the expression of genes involved in inflammation and oxidation mechanisms and increasing three-fold the expression of genes related to protection against oxidative stress. In addition, we found that this drink augmented antioxidant enzyme activity (17.8 vs. 8.2 nmol/mg), and prevented lipid peroxidation in the brain (49.7 vs. 96.5 nmol/mg). Therefore, we propose supporting the use of this drink by the general population as a new and global strategy for the prevention of neurodegeneration.

## 1. Introduction

Neurodegenerative diseases (ND), including Parkinson’s disease (PD) [1] and Alzheimer’s disease (AD) [2], are age-dependent disorders whose prevalence is rising due to the increasing life span of the world’s population [3]. According to the World Health Organization, the number of people with dementia worldwide was 46.8 million in 2015 but is expected to rise to 131.5 million by 2050 [4]. Dementia is the main symptom of NDs, and AD represents approximately 60–70% of all dementia cases [5,6]. ND are characterized by progressive loss of selectively vulnerable neuron populations in specific brain areas [7]. Unfortunately, nowadays, all these diseases lack an effective treatment and they represent the fourth cause of global disease burden in developed countries [8]. 

Alzheimer’s disease is defined by the progressive loss of short- and long-term memory, which results in an increasing cognitive deficit that leads to impaired activities of daily living [9]. Several factors including aging, diabetes mellitus, and oxidative stress, affect the risk of developing NDs [5,10]. On the other hand, decreased risk of AD is related with physically and cognitively stimulating activities and adherence to the Mediterranean diet [11]. Current literature supports that oxidative stress is one of the main risk factors for AD [12] and the reason for this is brain physiology. The brain, as the most oxygen-consuming organ in the human body, is easily damaged by oxidative stress, a variety of free radicals, and redox-active metals [13].

In recent years, there has been increasing supporting evidence for an association between lifestyle habits, such as diet and dietary components, and a delay in AD occurrence [14]. Functional foods, such as mushrooms, and drinks, such as red wine, exhibit potent medical properties with anti-oxidative and anti-inflammatory attributes [15,16]. Polyphenols encompass a large class of compounds (curcumin, stilbenes, flavonoids, etc.) with demonstrated antioxidant properties that are present in most vegetables and fruits. Since polyphenols are highly antioxidative in nature, their consumption may provide protection against neurological disorders [17]. More specifically, flavonoids are a type of polyphenols with demonstrated beneficial health effects derived from their antioxidant and anti-inflammatory properties [18]. 

Some studies show that flavonoid intake can have a protective effect on human and animal brains [19]. Recently, our group has described that oral administration of flavonoids, extracted from red and white grapes and from the olive tree, reduces the expression of brain genes involved in inflammation and oxidation mechanisms, whereas it increments the expression of Nrf2, a gene related to protection against oxidative stress. In the same way, preventive treatment with these natural flavonoids increases the activity of antioxidant enzymes and prevents lipid peroxidation in the brain of stressed mice [20].

The consumption of fruit and vegetable juices containing high concentrations of flavonoids, at least three times per week, may delay the onset of AD [21]. Although doubts were previously raised about flavonoids usefulness as beneficial antioxidant compounds, today we know that the vast majority of antioxidant substances need to be to be hydrolyzed by the small intestine mucosa or fermented by the colon microbiota prior to absorption [22]. For example, enzymes of the gut microbiota carry out modifications of flavonoids that result in smaller products, which may be easily absorbed in the gut [23]. Therefore, the bioavailability of flavonoids is guaranteed when orally administered [24,25].

The purpose of the present study was to develop a customer-acceptable drink with enriched antioxidant properties that could be used as a new and affordable strategy for the prevention of NDs. 

## 2. Materials and Methods

### 2.1. Natural Extracts and Grape Juice

Two commercial natural food supplements were used in this study. They included red grape polyphenols (generously provided by Alvinesa Natural Ingredients, Daimiel, Ciudad Real, Spain) and MecobalActive^®^ (generously provided by HealthTech Bio Actives, Barcelona, Spain).

Red grape polyphenols extracts from Alvinesa Natural Ingredients are entirely constituted by phenolic compounds (premium selected blending of monomers, dimers, oligomers, and polymers) and have a unique formulation that ensures direct absorption in the small intestine. These extracts are currently used as commercial supplements approved for human consumption. Red grape polyphenols extracts from Alvinesa are not only interesting because of the flavonoids of low molecular weight (monomers) but also because the substantial content in PACs. Alvinesa’s extracts present a PACs content (Porter Method) with a content 20–25% higher in comparison to our competitors (75% vs. 100%). Furthermore, Alvinesa is putting a lot of efforts in offering to the market the most natural extracts coming from grape producing extracts that are constituted 100% by polyphenols of the grape.

Grape juice was generously provided by Vintae winery (Logroño, Spain).

### 2.2. Preparation of MecobalActive^®^- and Red Grape Polyphenol-Containing Grape Juice

MecobalActive^®^ and red grape polyphenols were added at three different concentrations to the grape juice following previous results published by our group (Table 1) [20]. To get this, MecobalActive^®^ and red grape polyphenols were diluted in distilled water (2.0 g/L) using a magnetic stirrer (N-Agimatic, JP SELECTA, Abrera, Barcelona, Spain) at 1400 rpm for 1 min. The solution was added to the grape juice to obtain the different concentrations (0.375 mL/L, 0.6 mL/L, and 0.75 mL/L) and mixed with ultra-turrax (model T25, IKA Process, Staufen, Germany) at 6500 rpm for 1 min. Once mixed, the grape juice was bottled in 330 mL glass bottles and sealed with metal caps. 

### 2.3. Pasteurization Process

**Figure 1:**
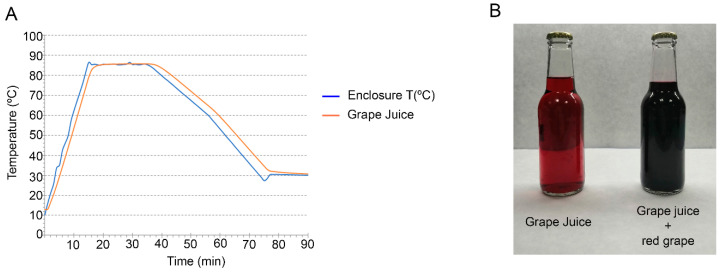
Temperatures in pasteurization process (**A**). Photograph of experimental bottles containing grape juice and grape juice with red grape polyphenols (**B**).

Once the samples were bottled, they were pasteurized to ensure shelf-life stability [26]. Two temperature probes (model Tracksense Pro, ELLAB AS, Hilleroed, Denmark) were used to monitor the pasteurization process: one to monitor the autoclave chamber temperature while another was inserted through the cap in a bottle to monitor juice temperature during the process. The pasteurization process took place in an autoclave (model APR-95, SURDRY, Abadino, Bilbao, Spain) and the process had three steps: (i) the samples were heated for 15 min. at 85 °C with an internal pressure of 1 bar; (ii) the samples were maintained at 85 °C for 20 min at 1 bar; and (iii) the samples were cooled at 25 °C for 40 min at 1 bar (Figure 1A). 

Pasteurization effect was calculated following the equation

$$P_{0}=\int_{0}^{t}LT\;dt$$

where:

*P*_0_ = Pasteurization value at 70 °C for *S. aureus,* which z parameter is 10 °C

*LT* = *S. aureus* lethality at 70 °C

*dt* = Time applied to each lethality

The final pasteurization effect was *P*_0_ = 915.3. Once pasteurized, bottles were kept in cold storage at 3 °C before analysis, tasting, and organoleptic evaluation.

### 2.4. Determination of Total Vitamin B12 Content

Total vitamin B12 content was determined by liquid chromatography/UV detection with immunoaffinity extraction, as previously described [27]. Briefly, Vitamin B12 is extracted in sodium acetate buffer in the presence of sodium cyanide (100 °C, 30 min). After purification and concentration with an immunoaffinity column, vitamin B12 is determined by liquid chromatography with UV detection (361 nm). Total vitamin B12 content was expressed as micrograms of vitamin B12 per 100 g.

### 2.5. Determination of Total Polyphenols Content

Total phenolic content was determined spectrophotometrically by Folin–Ciocalteu’s assay using gallic acid as standard, as previously described [28]. Briefly, 1.5 mL of gallic acid aqueous solutions or diluted grape juice or grape juice with red grape polyphenols, were added to 2.25 mL of methanol, and 1.5 mL Folin–Ciocalteu’s reagent, previously diluted in water (1:10, *v*/*v*). The mixture was shaken and allowed to stand for 5 min in the dark. Then, 1.5 mL of 7.5% (*w*/*v*) sodium carbonate solution was added to the mixture, and the reaction was kept in the dark for 30 min. After incubation, the absorbance was measured at 760 nm, versus the blank, in a spectrophotometer (Jenway, Staffordshire, UK). A blank was prepared, replacing gallic acid or grape juice by distilled water. Total phenolic content was expressed as milligrams of gallic acid equivalents (GAE) per gram.

### 2.6. Participants and Testing Location

This study was conducted at CTIC-CITA food technology center during 2020. To carry out the organoleptic evaluation, a tasting panel of six judges was set up based on UNE and ISO standards. The expert panel of CTIC-CITA has been established since 2009 and works on the sensory evaluation of different foods. Judges perform sensory tests to establish acceptance of future consumers and included tests for the five senses. 

All judges received a basic training based on the detection, recognition, and ranking of basic flavors, smells, and textures, through tests for the investigation of taste sensitivity and the recognition of thresholds of detection, identification, and differentiation according to the UNE-EN ISO 8586:2014 and the EN ISO 5492:2010 standards. Moreover, judges also received specific training based on the methodology for establishing an olfactory and gustatory profile according to the UNE-ISO 5496:2007 and the UNE-ISO 13302:2008 standards.

Testing took place in a private, comfortable room designed under UNE standards in CTIC-CITA in Alesón (La Rioja, Spain). Samples were analyzed organoleptically and a comparative study was carried out. The samples were evaluated to understand the critical organoleptic parameters of each product studied. Subjects consumed no food or drink other than water for at least one hour before the task and acclimated to the testing room and to the researcher for approximately 15 min. Judges evaluated the samples in a blind fashion. Samples were tasted in a sequential monadic order, one by one, in different order for each taster, following a randomized block design, to minimize the sample effect.

### 2.7. Testing Procedure

The samples and the control (original grape juice) were stored at 3 °C and served after homogenization. Two different series of grape juice were analyzed, following accepted methodology [29]. Each series included a different natural bioactive extract, MecolbalActive^®^ or red grape extract, and three different concentrations of each extract were tested (Table 1) in three different session days.

#### 2.7.1. Descriptive Analysis

Yes/No questions were asked to compare the similarity of the samples with the control taking into account the appearance, intensity of color, taste, and texture. 

#### 2.7.2. Assessment of Acceptability

For each sample, the appropriate score was selected by the judges according to the following range:

0—NOT ACCEPTABLE (outside organoleptic quality standards) 

5—ACCEPTABLE (within organoleptic quality standards)

10—VERY ACCEPTABLE (above organoleptic quality standards)

The mean value of all judgements was calculated. Samples with scores below 5 do not meet organoleptic quality standards according to UNE-ISO 8587:2010.

#### 2.7.3. Testing Preference

This method assesses the preference of certain attributes, characteristics, or overall valuation. This analysis evaluates the sample preferences on an ordered scale for each attribute, as specified in UNE-EN ISO 5495:2007 and UNE-ISO 8587:2010 standards. In this case, each of the samples was sorted according to its greater or lesser similarity with the control. The results are presented as a ranking scale of three scores in preferences from “the least” (1) to “the most” (3).

### 2.8. Restrain Stress and In Vivo Treatments

All procedures involving animals were carried out in accordance with the European Communities Council Directive (2010/63/EU) and Spanish legislation (RD53/2013) on animal experiments and with approval from the ethical committee on animal welfare of our institution (Órgano Encargado del Bienestar Animal del Centro de Investigación Biomédica de La Rioja, OEBA-CIBIR, procedure number AMR14).

Six-week-old C57BL/6J mice (Charles-River) were used for this assay. Mice were housed under standard conditions at a temperature of 22 °C (±1 °C) and a 12-h light/dark cycle with free access to food and water. 

Mice were subjected to an acute model of stress by immobilization, as previously described [20,30], by placing them inside 50 mL conical tubes with no access to food or water for 6 h. Adequate ventilation was provided by several air holes (0.5 cm in diameter) drilled into the conical end of the tube and at its sides. The tubes prevented forward, backward, or rotational movements of the mice. Due to the corticosterone circadian rhythm, restrain stress was applied at the same time of the day (9:00 AM) in all experiments. 

Mice were randomly divided into different experimental groups (*n* = 5 per group) and received, by oral gavage, 200 µL of grape juice with or without the natural extract (red grape) during 5 consecutive days. 2.8 mg of the natural extract were provided in each session. This amount was calculated as the mouse dose equivalent to a human dose of 180 mg/day following the recommendations of the Food and Drug Administration criteria for converting drug equivalent dosages across species (http://www.fda.gov/cber/gdlns/dose.htm (accessed on 5 April 2021) [31]. On the 6th day, mice were subjected to 6 h of restraint stress and immediately sacrificed. The whole brain was dissected out. The olfactory bulbs and the cerebellum were removed, and the remaining tissue was divided into two equal halves by a sagittal section. Each half was frozen separately in liquid N_2_ and stored at –80 °C. One side was used for RNA extraction and the other one for antioxidant enzymes analysis (see below). 

### 2.9. Quantitative Real-Time PCR

Total RNA was isolated from mouse brains and purified as described [32]. Briefly, total RNA was isolated using Trizol reagent (Invitrogen, Waltham, MA, USA) with DNAse digestion step performed (Qiagen, Hilden, Germany), according to manufacturer’s instructions. Resulting RNA (5 µg) was reverse transcribed using Superscript III First-Strand Synthesis System for RT-PCR (Invitrogen), and the synthesized cDNA was amplified using SYBR Green PCR Master Mix (Applied Biosystems, Foster City, CA, USA). Transcripts were amplified by real-time PCR (7300 Real-Time PCR System, Applied Biosystems). For each transcript, a specific calibration curve of cDNA was included to analyze expression of NOX-2, HMOX-1, IL-6, TNF-alpha, and Nrf-2. All measurements were normalized to GAPDH as a housekeeping gene. Specific primers are shown in Table 2.

### 2.10. TBARS, SOD, and Catalase Activity

For the determination of oxidative stress parameters and antioxidant components in the brain, frozen tissues were homogenized in RIPA buffer (Thermo Fisher Scientific, Waltham, MA, USA) supplemented with Complete and Phospho STOP (Roche, Basel, Switzerland) protease and phosphatase inhibitors as described [20]. Lipid peroxidation was determined using a commercial TBARS assay kit (CA995, Canvax, Cordoba, Spain). The final malondialdehyde products were detected by fluorescence spectroscopy with excitation/emission at 530 nm/590 nm in a microplate reader (POLARstar Omega, BMG Labtech, Ortenberg, Baden-Wuerttemberg, Germany). Levels of superoxide dismutase (SOD) activity were determined using an SOD assay kit (CA061, Canvax, Córdoba, Spain), according to the manufacturer’s protocol. Absorbance at 450 nm was measured using a POLARstar Omega plate reader. Catalase activities were determined using a commercial Catalase Activity assay kit (CA063, Canvax, Córdoba, Spain) following manufacturer’s instructions. Enzyme activity was detected by fluorescence spectroscopy with excitation/emission at 530 nm/590 nm in a microplate reader (POLARstar Omega).

### 2.11. Statistical Analysis

All data sets were analyzed for normalcy and homoscedasticity. Normal data were analyzed by Student’s *t* test or by One-way ANOVA followed by Dunnet’s multiple comparison post hoc test. Data that did not follow a normal distribution were compared by Kruskal–Wallis test followed by Mann–Whitney post-hoc test. Analyses were performed with GraphPad Prism version 8.3.0 (GraphPad Software, San Diego, CA, USA). A *p* value < 0.05 was considered statistically significant.

## 3. Results

### 3.1. Vitamin B12 and Polyphenol Levels Are Not Negatively Affected by Grape Juice Pasteurization

The purpose of this study was to develop a customer-acceptable food staple with enriched antioxidant properties that may be used to prevent NDs. Grape juice was chosen as the alimentary matrix and different natural extracts that had previously demonstrated antioxidant capacity in vivo [20] were added. Extracts selected for this study were MecobalActive^®^ and red grape polyphenols. Three different concentrations of the extracts were established (Table 1) based on our previous published experience [20] and pasteurization of the juice was performed as a strategy to preserve and standardize the drink. To determine whether pasteurization negatively affected stability of the bioactive extracts, physicochemical studies were carried out to determine the amount of vitamin B12 and polyphenols present in the grape juice before and after pasteurization. The study was done for the higher (Mec3 and Gra3) and lower (Mec1 and Gra1) extract concentrations. 

In the case of vitamin B12, the original grape juice contained no detectable amount of vitamin B12 (Table 3). Two enriched samples were prepared, containing 75 and 150 µg of MecolActive^®^ per 100 g of grape juice, and were named Mec1 and Mec3, respectively. Physicochemical studies were carried out after pasteurization showing that only 67 and 117 µg of MecolActive^®^ remained in the Mec1 and Mec3 samples, respectively. This study demonstrated that pasteurization results in the loss of between 10.7% and 22.0% of vitamin B12 (Table 3). Nevertheless, this loss was not considered problematic since the remaining levels of vitamin B12 are sufficient to elicit a beneficial effect in vivo.

In the case of polyphenols, the original juice contains 346.4 mg/kg (Table 4). As with vitamin B12, two samples were prepared by adding 500.0 and 1250.0 µg/Kg of red grape polyphenols to the grape juice (named as Gra1 and Gra3, respectively). Physicochemical studies carried out after pasteurization showed that only 601.3 and 1124.0 µg/Kg were present in Gra1 and Gra3, respectively. These results represent a loss of 28.96% and 29.59% on polyphenol contents in Mec1 and Mec3 respectively, due to pasteurization (Table 4). As with vitamin B12, this loss was not considered relevant since the remaining polyphenol levels were sufficient to induce healthy in vivo effects.

### 3.2. MecobalActive^®^ Addition to the Grape Juice Does Not Cause Significant Changes in Its Aspect, Texture, and Color and Odor Intensities

After demonstrating that vitamin B12 and polyphenol levels were stable in the pasteurized drink, a comparison of organoleptic characteristics was performed between the untreated control (grape juice) and the different enriched samples by conducting a sensorial analysis.

First, the impact of adding three different concentrations of MecobalActive^®^ to the grape juice was studied by a panel of selected judges. Taking into account the aspect, 83.3% of judges considered that all MecobalActive^®^ concentrations (Mec1, Mec2, and Mec3) had no differences compared to the control. Regarding color and odor intensity, 83.3% considered that Mec1 and Mec2 were equal to the control, whereas for Mec3 50.0% of judges decided that differences with the control were evident. For taste, 66.7% of judges considered samples Mec1 and Mec3 equal to the control, but for Mec2 only 33.3% decided that it was undistinguishable from the control. Taking into account the flavor, 50.0% of the panel considered that samples Mec1 and Mec2 were equal to the control but, in contrast, sample Mec3 was different from the control according to 83.3% of judges. Finally, regarding texture, 83.3% of judges considered that Mec1 and Mec2 were equal to control but only 50.0% of the panel decided that Mec3 was equal to control.

### 3.3. The Addition of Red Grape Polyphenols Does Not Cause Relevant Changes in the Intensity of Flavor and Texture of the Grape Juice

The addition of three different concentrations of red grape polyphenols to the grape juice was studied by the same panel of judges. Taking into account the aspect, and color and odor intensity, 100% of judges considered that all modifications (Gra1, Gra2, and Gra3) were different from the control. The differences in color are evident due to the strong hue of the extracts (Figure 1B). For taste, 50% of judges considered only sample Gra1 equal to the control. Regarding flavor, 50% of the panel considered that Gra1 and Gra2 were equal the control but, in contrast, sample Gra3 was not equal to the control according to 66.6% of judges. Finally, concerning texture, 100% of judges considered that Gra1 was equal to control and half of the panel thought the same thing for Gra2 and Gra3.

### 3.4. MecobalActive^®^ and Red Grape Polyphenols Addition to the Grape Juice Does Not Cause Variation in Its Organoleptic Standards

**Figure 2:**
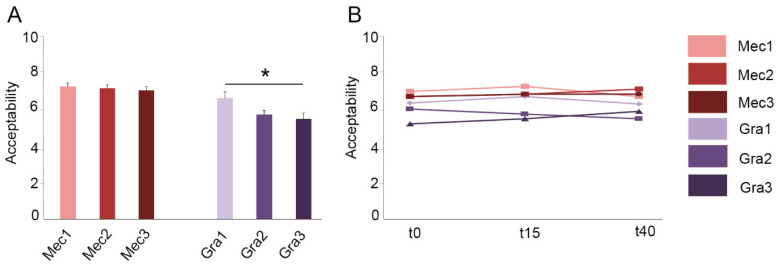
Acceptability test for all samples measured at time 0 (**A**) and evolution over a period of time (**B**). Samples indicate different concentrations of the active ingredients as shown in Table 1. Values are presented as mean ± SEM from at least eighteen independent measures. Kruskal-Wallis and Dunn’s multiple comparison post hoc test were used for statistical analysis. * *p <* 0.05.

The organoleptic quality of the samples was evaluated by the judges using the acceptability test. Samples enriched with MecobalActive^®^ obtained values between 7.17 and 6.94, with no statistically significant differences among them (Figure 2A). However, significant differences were obtained among the three concentrations of red grape polyphenols, where Gra1 showed the highest acceptability, at 6.52 (Figure 2A). All samples obtained values above 5.0, indicating that they were within organoleptic acceptable standards. In addition, the evolution of acceptability was monitored to study whether the organoleptic properties were lost over time. No difference in the organoleptic standards was observed for any of the samples up to 40 days after sample preparation (Figure 2B), indicating that the organoleptic quality of the samples does not change over this time period.

### 3.5. The Sample with the Lowest Concentration of Red Grape Polyphenols Shows Higher Preference by the Judges

**Figure 3:**
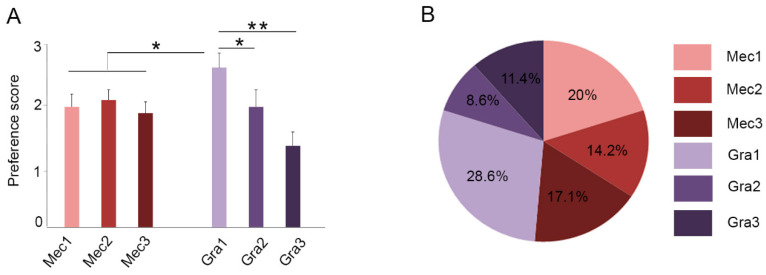
Ranking (**A**) and greater acceptability (**B**) tests for the six samples of the study. Samples indicate different concentrations of the active ingredients as shown in Table 1. Values are presented as mean ± SEM from at least 18 independent measures. Kruskal-Wallis and Dunn’s multiple comparison post hoc test were used for statistical analysis. * *p <* 0.05; ** *p <* 0.01.

Since the purpose of the study is to develop a customer-acceptable drink with enriched antioxidant properties, and given that all samples were within optimal organoleptic standards, a ranking test was conducted to select the best presentation. This test asks the judges to score the samples with a ranking scale of their preferences from “the least” (one point) to “the most” (three points) favorite drink. According to the order given by each judge, the average score value was calculated and a general ranking was established for each sample (Figure 3A). Gra1 was the sample with the highest score (2.64 ± 0.24) compared to the other presentations (*p* < 0.05) (Figure 3A). 

Finally, judges were asked to select the single sample with greater acceptability. The sample that showed greater acceptability among judges was Gra1 (28% of judges), followed by Mec1 (20%), Mec3 (17.1%), Mec2 (14.2%), Gra3 (11.4%), and Gra2 (8.6%) (Figure 3B). Taking into account all previous results, we selected Gra 1 (red grape polyphenols at lowest concentration) as the best sample to carry out in vivo studies.

### 3.6. Oral Administration of Grape Juice Supplemented with Red Grape Polyphenols Exerts an Antioxidant Effect in the Brain of Stressed Mice

Previous studies have demonstrated that red grape polyphenols possess in vivo antioxidant capacity [20] but the presence of the alimentary matrix (grape juice) may interfere with this property. Therefore, grape juice with or without added red grape polyphenols was orally administered for five days and then mice were subjected to immobilization stress for 6 h. Expression of inflammatory and oxidative genes was studied by qRT-PCR in the mouse brains.

**Figure 4:**
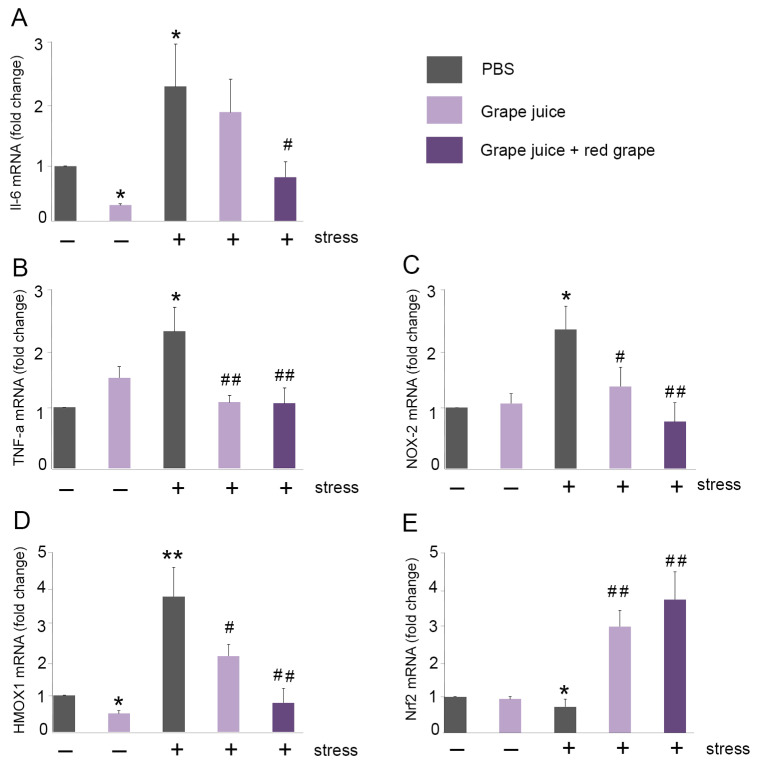
Grape juice enriched with red grape polyphenols protects against oxidative stress. Grape juice with or without red grape extract was administered orally during 5 consecutively days. Mice were immobilized for 6 h. mRNA expression of IL-6 (**A**), TNF-alpha (**B**), NOX-2 (**C**), HMOX1 (**D**), and Nrf2 (**E**) was quantified in mouse brains by qRT-PCR. Gene expression was normalized with GAPDH. All data were normalized to levels found in non-stressed mice and are expressed as fold change. Values are presented as mean ± SEM from at least five independent animals. One-way ANOVA and Dunnet’s multiple comparison post hoc test were used for statistical analysis. * *p <* 0.05; ** *p <* 0.01 vs. normal mice; ^#^ *p* < 0.05; ^##^ *p* < 0.01 vs. stressed mice.

To mimic the human dose provided in Gra1 (180 mg/unit), mice received 200 µL of grape juice containing 2.8 mg of red grape polyphenols, according to published conversion standards [31]. In agreement with our previous results [20], immobilization stress significantly increased the expression of IL-6 and TNF-alpha when compared to control (two-fold and 2.5-fold, respectively) (Figure 4A,B). Administration of grape juice resulted in a diminution of the expression of both genes that was statistically significant for TNF-alpha (Figure 4A,B). Furthermore, grape juice enriched with red grape polyphenols returned IL-6 and TNF-alpha expression to values undistinguishable from those obtained in the non-stressed animals (Figure 4A,B). We also studied the expression of NOX-2 (Figure 4C) and HMOX-1 (Figure 4D). These genes are involved in oxidation mechanisms and they increase in the brain of mice subjected to stress [30]. The administration of red grape polyphenols-enriched grape juice significantly decreased the immobilization-increased expression of both NOX-2 and HMOX-1 to levels very similar to those found in the animals without stress (Figure 4C,D). Finally, we analyzed Nrf-2 expression (Figure 4E). Numerous authors have described Nrf-2 expression as a protective mechanism for oxidative stress [33,34,35]. In ours case, immobilization stress significantly reduced Nrf-2 expression (Figure 4E) but administration of grape juice with or without the natural extract significantly increased Nrf-2 expression to high levels (Figure 4E).

### 3.7. Preventive Treatment with Grape Juice Enriched with Red Grape Polyphenols Increases Antioxidant Enzymes Activity in the Brain

To verify the possible protective role of red grape polyphenol-enriched grape juice in oxidative stress, we studied the activity of two antioxidant enzymes, catalase and superoxide dismutase (SOD), in the mouse brains.

**Figure 5:**
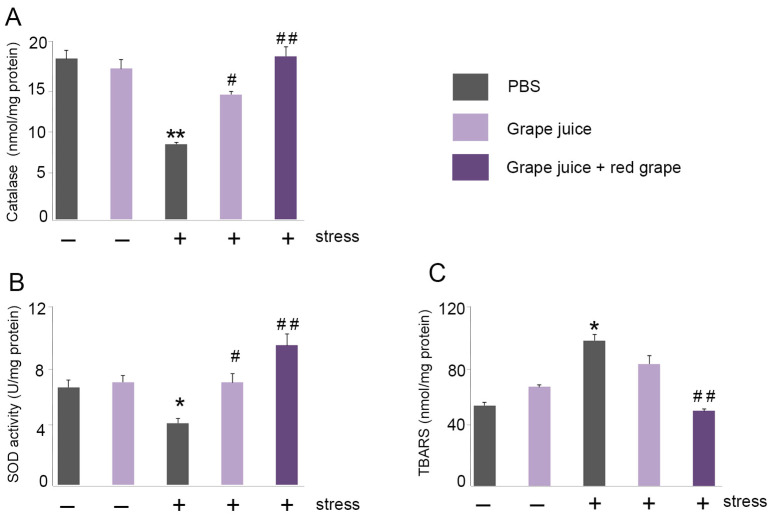
Grape juice enriched with red grape polyphenols increases the activity of antioxidant enzymes. Mouse brains were isolated and catalase activity (**A**), SOD activity (**B**), and TBARS (**C**) were analyzed. Values are presented as mean ± SEM from at least five independent animals. One-way ANOVA and Dunnet’s multiple comparison post hoc test were used for statistical analysis. * *p <* 0.05; ** *p <* 0.01 vs. normal mice; ^#^ *p* < 0.05, ^##^ *p* < 0.01 vs. stressed mice. Abbreviations: SOD: superoxide dismutase.

In a previous report, we described that stress causes a decrease in catalase activity in the mouse brain [30]. Indeed, we observed a significant reduction in catalase activity in stressed mice compared to non-stressed animals (Figure 5A). Furthermore, the administration of grape juice with or without red grape polyphenols led to a statistically significant increase in the levels of catalase activity (Figure 5A). SOD is one of the most important antioxidant enzymes in the cells catalyzing the dismutation of the superoxide anion into hydrogen peroxide and molecular oxygen [36]. As with catalase activity, stress caused a significant decrease in SOD activity in the mouse brains (Figure 5B). Interestingly, the administration of grape juice with or without red grape polyphenols significantly increased the activity of SOD enzyme (Figure 5B). The expression was higher in the case of grape juice with red grape polyphenols, indicating their potent antioxidant effect (Figure 5B).

### 3.8. Preventive Treatment with Red Grape Polyphenol-Enriched Grape Juice Prevents Lipid Peroxidation in the Brain

Lipid peroxidation is another important parameter to take into account when studying oxidative stress [37]. We measured malondialdehyde (MDA) levels present in the mouse brain. In a previous report, we observed that acute stress doubled MDA levels when compared with the non-stressed control group [30]. First, we confirmed this elevation in MDA due to immobilization stress (Figure 5C). Treatment with grape juice alone did not provide a significant protection, but grape juice enriched with red grape polyphenols resulted in a significant reduction in MDA levels (*p* < 0.01). The MDA levels of animals treated with the enriched juice were undistinguishable from those found in the animals without stress (Figure 5C).

## 4. Discussion

Here, we have developed a customer-acceptable drink with enriched antioxidant properties and we have examined its protective properties against oxidative stress in vivo. We found that the addition of red grape polyphenols and MecobalActive^®^ to grape juice did not provoke changes in juice organoleptic characteristics and that the pasteurization process did not destroy the levels of flavonoids and vitamin B12 present in the grape juice. A panel of judges selected grape juice with red grape polyphenols and it was used to carry out in vivo studies. In vivo, we demonstrated that oral administration of grape juice supplemented with red grape polyphenols exerted an antioxidant effect in the brain of stressed mice reducing the expression of genes involved in inflammation and oxidation mechanisms and increasing the expression of genes related to protection against oxidative stress. In addition, we found that preventive treatment with grape juice enriched with red grape polyphenols augmented antioxidant enzymes and prevented stress-induced lipid peroxidation in the brain.

NDs are age-dependent disorders whose prevalence is rising due to the increasing life span of the world’s population. In recent years, there has been increasing supporting evidence for an association between lifestyle habits, such as diet and dietary components, and a delay in AD occurrence [14] and, specifically, some studies show that flavonoid intake can have a protective effect on human and animal brains [19,20] and that the bioavailability of flavonoids is guaranteed when orally administered [24,25]. The vast majority of antioxidant substances need to be fermented by the microbiota of either the small intestine or the colon to achieve optimal absorption [24,25].

Some studies have shown that moderate wine consumption, specifically red wine, could have a neuroprotective effect due to the fact that grapes are one of the richest sources of polyphenols [14]. In this study, we were interested in using a non-alcoholic drink. It has also been described that the consumption of fruit and vegetable juices containing high concentrations of flavonoids, at least three times per week, may delay the onset of AD [21]. Given all this, grape juice was chosen as the drink to use in the study. Moreover, red grape polyphenols and MecobalActive^®^ were selected as the extracts to use in the study because they showed great results in an in vivo restraint stress model which was recently published by our group [20].

Heat treatments, such as pasteurization and sterilization, are the most used methods to process and preserve food, mainly due to their ability to inactivate a wide range of microorganisms and spoilage enzymes [38]. However, heat processing may induce several chemical and physical changes, reducing the content and, also, the bioavailability of some bioactive compounds such as polyphenols [39,40]. Physicochemical studies carried out in grape juice with MecobalActive^®^ or red grape polyphenols after pasteurization showed that a loss of about 20–30% of vitamin B12 and polyphenol contents took place due to the heat process. This fact was not a surprise because many papers describing heat damage to polyphenols availability have been published. Losses of between 16 and 28% of polyphenol levels after pasteurization have been previously reported in grape and watermelon juice and strawberry puree [41,42,43,44]. The really important fact is that this loss is not considered relevant, since the remaining polyphenols are sufficient to induce healthy in vivo effects [45].

In the sensorial study, panelist noted no significant changes in the aspect, texture, and color and odor intensities on grape juice supplemented with MecobalActive^®^, whereas the addition of red grape polyphenols did not cause relevant changes in the intensity of flavor and texture of the grape juice. In 2014, Barba et al. [46] proposed that changes in sample color are noticeable by an inexperienced observer if they are higher than 1.5-fold. Here, an expert panel of judges determined that there were no significant changes in grape juice color after adding MecobalActive^®^ although there was an evident change after the addition of red grape polyphenols since they provide an intense purple coloration. In addition, the panelists only expressed a significant dislike for the sample with the higher concentration of red grape polyphenols, although the sample met the acceptability requirements dictated by UNE-ISO 8587:2010. Finally, the sample best considered by the judges in both acceptability and ranking was grape juice with the lowest concentration of red grape polyphenols and, for this reason, this sample was chosen to carry out in vivo studies in animals. However, it should not be forgotten that grape juice supplemented with MecobalActive^®^ also achieved optimal acceptability values by the judges and could be a future candidate to test in vivo.

The use of stress models is supported by substantial evidence implicating stress as a precipitating factor for several neuropsychiatric disorders [47]. We and others have used acute restraint stress with 6 h of immobilization for our stress-inducing experiments achieving satisfactory results [30,48]. Acute restraint stress stimulates several cellular events resulting in enhanced ROS production [49]. Furthermore, the extracellular release of ROS finally enhances production of pro-inflammatory cytokines IL-1β, IL-6, and TNF-α [50,51]. In addition, Nox-2 is well known for generating superoxide molecules under oxidative stress-mediated circumstances and HMOX1 is induced by oxidative stress [52]. On the other hand, Nrf2 induces the expression of antioxidant genes which eventually provoke an anti-inflammatory expression profile that is crucial for the initiation of healing [53]. Our previous studies demonstrate that red grape polyphenols possess an important antioxidant capacity in vivo [20] but the presence of the alimentary matrix (grape juice) may interfere with this property. Here we described that the administration of grape juice with red grape polyphenols prevents the expression of genes involved in inflammation and oxidation mechanisms, while increasing the expression of Nrf2 confirming that the presence of the grape juice did not interfere with the antioxidant capacity of red grape polyphenols, but on the contrary, they may act synergistically. We described that grape juice alone reduced TNF-α and NOX-2 expression, and increased 2.5-fold Nrf2 levels. It is noteworthy that the grape juice by itself was able to partially correct some of the genetic parameters (TNF-α, NOX2, HMOX1, and Nrf2). This is in agreement with previous studies on the antioxidant properties of grape products [54]. This antioxidant capacity has also been reported for other juices. For instance, juices rich in vitamin C such as acerola, wild rose, and pompia juice have in vivo antioxidant capacities [55,56]. In addition, pomegranate juice can be used as an anti-oxidative and anti-inflammatory agent, as demonstrated in mouse models [57].

It is understood that ND are induced by chronic stress, but single experiences of acute stress may have long-term consequences on brain physiopathology [58]. This is due to the brain’s neuroplasticity. Recent studies have shown that acute or subacute stress can induce not only rapid, but also sustained changes in synaptic function, neuroarchitecture, and behavior [59]. In any case, it would be interesting to confirm our data on a model of chronic stress.

Similarly, numerous studies have reported that restraint stress enhances lipid peroxidation and decreases antioxidant enzyme activities in rodents [49,60]. SOD and catalase are the best-known antioxidant enzymes [30]. We found that grape juice supplemented with red grape polyphenols increased the activity of catalase and SOD when compared to stressed mice. On the other hand, MDA is one of the final products of polyunsaturated fatty acid peroxidation in the cells and is commonly used as a marker of oxidative stress [61]. In agreement with this, we found that MDA levels significantly increased in the brain of stressed animals but were very efficiently normalized by oral administration of the grape juice with red grape polyphenols. All these results allow us to confirm that the presence of the grape juice did not interfere whit the antioxidant capacity of red grape polyphenols.

## 5. Conclusions

Taken together our results suggest that the addition of red grape polyphenols to grape juice is well tolerated by potential customers and that the new drink effectively reduces stress levels in the brain. In consequence, we propose that private companies and the public administration may collaborate in promoting the consumption of this drink, or similarly characterized products, by the general public as a new and global strategy for the prevention of NDs.

## Figures and Tables

**Table 1:** Concentration of active extracts in each experimental sample.

Code	Natural Extract	Concentration
Mec1	MecobalActive^®^	0.25 mg/unit
Mec2	MecobalActive^®^	0.40 mg/unit
Mec3	MecobalActive^®^	0.50 mg/unit
Gra1	Red grape	180 mg/unit
Gra2	Red grape	350 mg/unit
Gra3	Red grape	500 mg/unit

**Table 2:** Primers used in this study. Annealing temperature was 60 °C for all transcripts.

Gene	Forward Primer (5′–3′)	Reverse Primer (5′–3′)	Accession Number
** NOX-2 **	GCTGGGATCACAGGAATTGT	CTTCCAAACTCTCCGCAGTC	NM_007807
** HMOX-1 **	TGCTCGAATGAACACTCTGG	TAGCAGGCCTCTGACGAAGT	NM_010442
** IL-6 **	ATGGATGCTACCAAACTGGAT	TGAAGGACTCTGGCTTTGTCT	NM_031168
** TNF-alpha **	CCACCACGCTCTTCTGTCTA	CACTTGGTGGTTTGCTACGA	NM_001278601
** Nrf-2 **	AGCGAGCAGGCTATCTCCTA	TCTTGCCTCCAAAGGATGTC	NM_010902
** GAPDH **	CATGGCCTTCCGTGTTCCTA	GCGGCACGTCAGATCCA	NM_008084

**Table 3:** Vitamin B12 levels in original grape juice, and in Mec1 and Mec3 samples.

	VitB12 (µg/100 g)		
	Grape Juice	Mec1	Mec3
Added amount		75	150
Expected amount		75	150
Obtained value	0	67	117
Lost amount		8	33
% loss		10. 7%	22.0%

**Table 4:** Polyphenol levels in grape juice, and in Gra1 and Gra3 samples.

	Polyphenols (mg/Kg)		
	Grape Juice	Gra1	Gra3
Added amount		500.0	1250.0
Expected amount		846.4	1596.4
Obtained value	346.4	601.3	1124.0
Lost amount		245.1	472.4
% loss		28.96%	29.59%

## Data Availability

The data presented in this study are available on request from the corresponding author.
